# Dual treatment of acromegaly and hormone-receptor-positive breast cancer with tamoxifen: a case report

**DOI:** 10.1186/s13256-021-02792-8

**Published:** 2021-04-29

**Authors:** Sasan Mirfakhraee, Alberto V. Cabo Chan, Niloofar Ganji, Jessica Abramowitz

**Affiliations:** 1 Division of Endocrinology and Metabolism, Department of Internal Medicine, University of Texas Southwestern Medical Center, Dallas, TX USA; 2 Division of Mineral Metabolism, Department of Internal Medicine, University of Texas Southwestern Medical Center, Dallas, TX USA; 3 Department of Physiology, University of Toronto, Toronto, ON Canada; 4 UT Southwestern Medical Center, WCB3 8th Floor, 2001 Inwood Rd, Dallas, TX 75390 USA

**Keywords:** Acromegaly, Pituitary tumors, Growth-hormone-secreting pituitary adenoma, Case report

## Abstract

**Background:**

Adjuvant endocrine therapy is recommended for the treatment of hormone-receptor-positive breast cancer. Aromatase inhibitors are associated with significant musculoskeletal adverse effects, likely through growth hormone/insulin-like growth factor 1 modulation, while tamoxifen reduces insulin-like growth factor 1 production. We describe the case of a patient who was treated successfully with tamoxifen for her hormone-receptor-positive breast cancer and acromegaly.

**Case presentation:**

A 57-year old White female with hormone-receptor-positive breast cancer was diagnosed with acromegaly. She received adjuvant endocrine therapy with anastrozole but could not tolerate this medication because of severe arthralgia, so she was switched to tamoxifen. Shortly after starting tamoxifen, the patient’s musculoskeletal symptoms resolved and her insulin-like growth factor 1 levels normalized. She has remained in remission of her acromegaly and breast cancer since initiating tamoxifen.

**Conclusion:**

This case highlights the dual benefit of tamoxifen therapy in the treatment of hormone-receptor-positive breast cancer and acromegaly. Unlike anastrozole, tamoxifen has the benefit of lowering insulin-like growth factor 1 levels, which underscores its advantage in reducing adverse musculoskeletal symptoms during the treatment of hormone-receptor-positive breast cancer. We offer the first reported use of tamoxifen monotherapy for the successful treatment of acromegaly and hormone-receptor-positive breast cancer. While tamoxifen may offer an additional, oral option for acromegaly patients who do not respond to or tolerate conventional growth-hormone-lowering therapy, additional studies are necessary.

## Background

Acromegaly is a chronic disease caused by growth hormone overproduction, most commonly by a pituitary adenoma, with characteristic bone and soft tissue changes. Individuals with acromegaly are subject to extensive comorbid illnesses and complications. An increase in neoplasia incidence with acromegaly remains controversial, but increased rates of breast cancer have been noted previously [1]. While on treatment with antineoplastic agents, mortality rates from cancer are higher in acromegaly patients whose growth hormone levels remain elevated, and those with reduced growth hormone levels have a mortality rate similar to the general population [2].

Hormone-receptor (that is, estrogen receptor [ER] and progesterone receptor [PR]) testing is recommended for all invasive breast cancers, as this predicts which patients may benefit from adjuvant endocrine therapy. Since ER-positive breast cancers portend an increased risk of late recurrence, recent American Society of Clinical Oncology (ASCO) clinical practice guidelines recommend extended adjuvant endocrine therapy with aromatase inhibitors (AI) or tamoxifen for 10 years total [3]. Unfortunately, AI are associated with significant musculoskeletal adverse effects, including bone pain and arthralgia in as many as 61% of users, with up to 20% of patients discontinuing therapy because of pain [4]. While several potential etiologies have been proposed for AI-induced arthralgia [5], an intriguing possibility involves growth hormone (GH)/insulin-like growth factor 1 (IGF-1) modulation by AI therapy with subsequent development of adverse musculoskeletal effects. AI therapy has been shown to result in increased IGF-1 levels and an increased incidence of arthralgia in women treated for breast cancer, while tamoxifen reduced IGF-1 levels and the overall incidence of musculoskeletal side effects when used as adjuvant therapy [6].

## Case presentation

A 57-year-old White female with past medical history of obstructive sleep apnea, nontoxic multinodular goiter, and hypertension underwent a routine mammogram, which revealed a focal asymmetry in the right breast; this was subsequently characterized on ultrasound as an irregular, hypoechoic solid mass with indistinct margins measuring 14 mm. A biopsy revealed an invasive ductal carcinoma, grade 1, ER 100%, PR 100%, HER2/neu 1+ with a Ki-67 index of 5%. The patient underwent partial mastectomy with sentinel node biopsy and subsequently received radiation therapy.

**Fig. 1:**
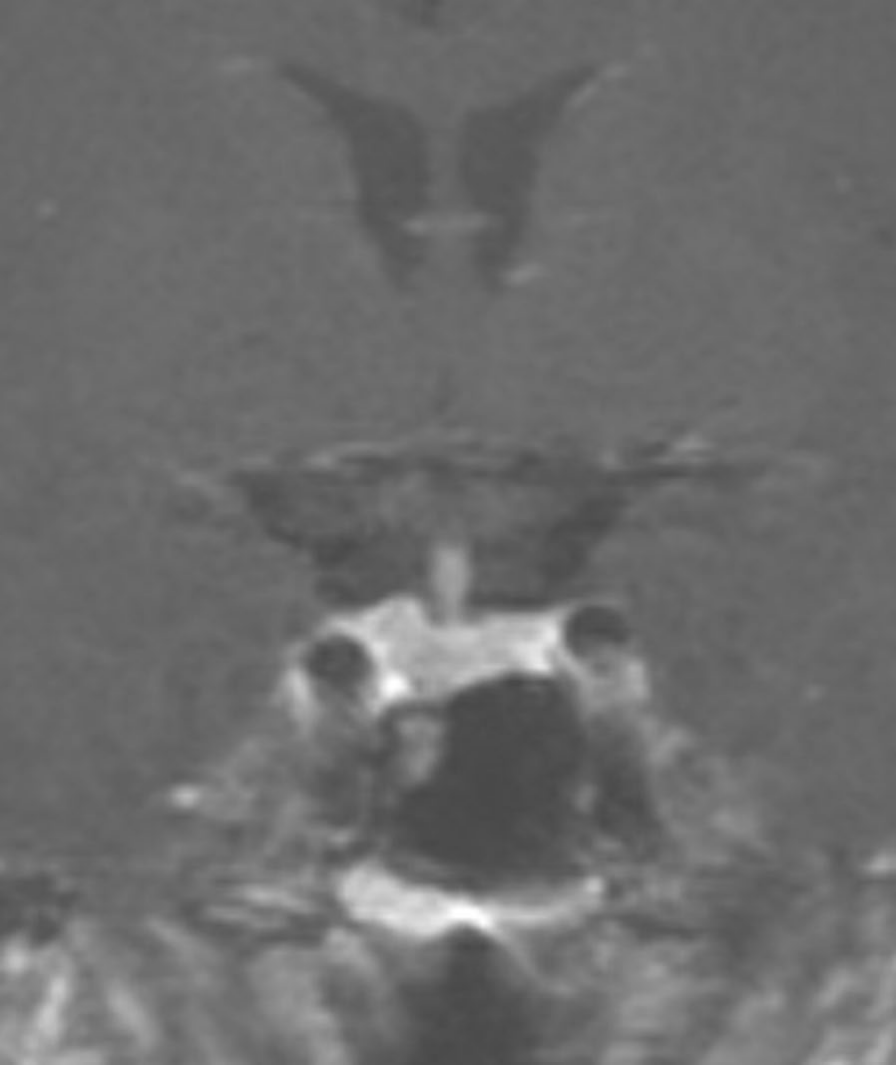
T1-weighted, dynamic 3T magnetic resonance imaging of the pituitary revealing subtle asymmetry involving the right aspect of the sella without focal lesion noted

The patient was then referred to our endocrine clinic for treatment of her osteopenia, when it was noted that she had features consistent with growth hormone excess, including frontal bossing, jaw protrusion, wide-spaced teeth, deep voice, diaphoretic palms, and enlarged hands and feet. Symptomatically, she noted episodic headaches and diaphoresis of the hands but denied arthralgia or change in ring, shoe, or hat size. Biochemical evaluation for growth hormone excess at this time revealed an IGF-1 level of 535 ng/mL by LC/MS (reference range 50–317 ng/mL, *Z*-score > 3) and morning fasting growth hormone level of 1.57 ng/mL (reference range 0.01–3.61 ng/mL). Two-hour oral glucose tolerance testing was performed, which showed failure of growth hormone to suppress, with a nadir level of 1.4 ng/mL. Dynamic 3 Tesla magnetic resonance imaging (3T MRI) of the pituitary with gadolinium administration revealed subtle asymmetry of the right aspect of the sella without discrete lesion noted (Fig. 1).

**Fig. 2:**
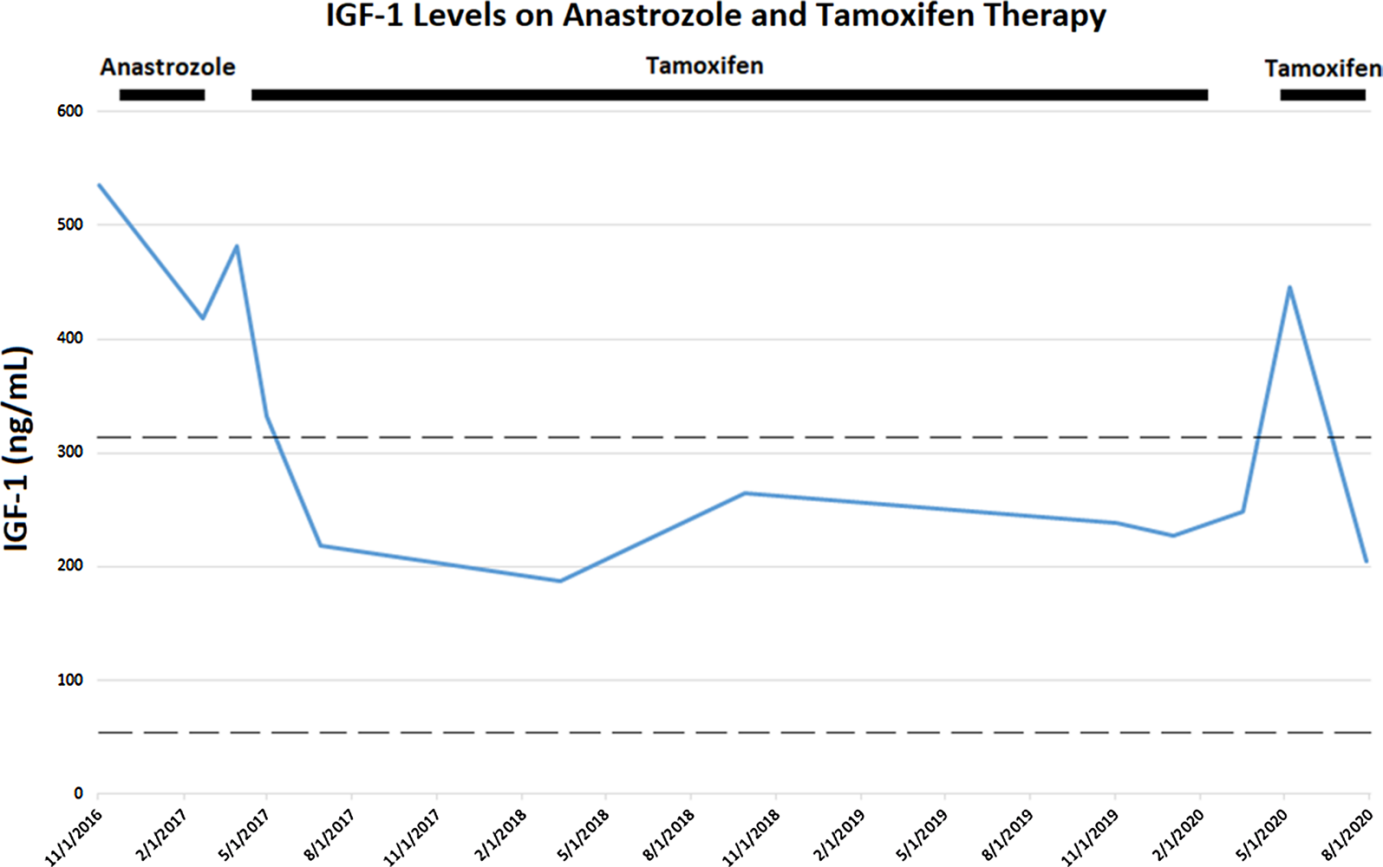
IGF-1 levels (LC/MS) before and after anastrozole therapy, then while on tamoxifen therapy. Upper- and lower-normal reference ranges (50–317 ng/mL) shown as dashed lines

The patient opted to address her malignancy first prior to consideration of definitive therapy of her acromegaly. She was started on adjuvant endocrine therapy with anastrozole for her breast cancer but developed severe arthralgia, so was changed to tamoxifen, with prompt resolution of her headache and musculoskeletal symptoms. Two months after tamoxifen initiation, the patient’s IGF-1 levels normalized and remained within the normal reference range for over 3 years while she continued tamoxifen treatment (Fig. 2). Her growth hormone levels ranged from 1.4 to 2 ng/mL while on tamoxifen therapy. Recently, the patient briefly discontinued the tamoxifen for 3 months to see if this was the cause of her diminished energy levels; her IGF-1 levels increased above the normal reference range from 249 to 446 ng/mL and her acral symptoms recurred, so she restarted tamoxifen, with successive normalization of her IGF-1 level to 205 ng/mL. Repeat imaging 3 months after restarting tamoxifen therapy again failed to reveal a discrete sellar lesion.

Given the patient’s excellent IGF-1 response to tamoxifen, she has opted for continued medical therapy rather than surgical exploration. When she completes her course of tamoxifen next year, she prefers to transition to oral octreotide for ongoing treatment of her acromegaly. Regarding her breast cancer, she has had no evidence of recurrence 4 years since the time of diagnosis.

## Discussion

Orally administered estrogen inhibits hepatic IGF-I synthesis through first-pass hepatic effect but increases GH secretion by reduced feedback inhibition [7]. This reduction in IGF-1 levels, despite an overall increase in circulating GH, is explained by an inhibitory effect on the metabolic action of GH mediated by oral estrogen. Oral estrogen therapy attenuates GH signaling by inducing suppressors of cytokine signaling 2 (SOCS-2), thereby inhibiting JAK2 phosphorylation through the JAK/STAT pathway [8]. Analogous to this effect of oral estrogen, selective estrogen receptor modulators (SERMs) act as an estrogen agonist on the liver [9] and reduce IGF-1 production. This gives them a unique advantage both in the treatment of hormone-receptor positive breast cancer as well as in acromegaly. In a study of 17 subjects (15 men, 2 postmenopausal women) with acromegaly whose IGF-1 levels remained elevated despite conventional, GH-lowering medical therapy, the addition of tamoxifen reduced IGF-1 levels by an average of 90 ng/mL and normalized plasma IGF-1 in 47% of subjects [10]. Tamoxifen also lowered IGF-1 levels in 13 out of 19 subjects with acromegaly (6 men, 13 women), normalizing IGF-1 levels in 21% of participants [11]. Tamoxifen does not lower GH levels in acromegaly, however, since GH secretion remains autonomous. In a study of 16 male patients with refractory acromegaly, despite conventional therapy, the addition of clomiphene citrate, a SERM, reduced IGF-1 levels by 41% but caused a nonsignificant increase in GH levels [12].

The GH/IGF-1 axis plays an integral role in breast development; furthermore, when GH and IGF-1 levels are experimentally perturbed, hyperplastic lesions may develop, increasing the chances of mammary carcinoma [13]. The IGF-1 receptor is implicated in breast cancer, with up to 50% of breast tumors expressing the activated form of this receptor [14]. Circulating IGF-1 levels are also associated with breast cancer risk, with pooled data from 17 prospective studies demonstrating a positive association between IGF-1 levels and estrogen-receptor-positive cancer risk [15]. Furthermore, higher IGF-1 levels are related to reduced overall survival in breast cancer patients treated with endocrine therapy [16]. Tamoxifen lowers plasma IGF-1 levels in individuals with breast cancer [17], which may contribute to its antitumor action. While interventions aimed at inhibiting the IGF-1R have not improved outcomes in breast cancer subjects to date, various elements of study design may be contributory to this outcome [18] and further studies are ongoing.

There are two cases in the literature describing cases of acromegaly and hormone-receptor-positive breast cancer in women [19, 20]. The first case details the response to breast cancer therapy in a woman with ER+/PR+ metastatic disease treated concomitantly with exemestane, an AI, and octreotide for her acromegaly; the subject’s breast cancer progressed when she was nonadherent with octreotide therapy [19]. In the second case, the IGF-1 levels of a patient with acromegaly remained elevated despite lanreotide and cabergoline therapy [20]. Shortly thereafter, the patient was diagnosed with ER+/PR− breast cancer. Tamoxifen was substituted for the patient’s cabergoline, with subsequent normalization of the patient’s IGF-1 levels. Our case differs from these two published cases in that our patient was not treated with conventional medical therapy for acromegaly; instead, she received initial therapy with anastrozole, which exacerbated her musculoskeletal symptoms, presumably mediated by the effect on her GH/IGF-1 axis. She was then switched to tamoxifen, which resolved her acral symptoms and normalized her IGF-1 levels. Ours is therefore the first-reported use of tamoxifen monotherapy for the successful treatment of acromegaly and hormone-receptor-positive breast cancer.

## Conclusion

We report the case of a patient with hormone-receptor-positive breast cancer who was diagnosed with acromegaly prior to the initiation of adjuvant endocrine therapy. Her symptoms worsened on anastrozole but improved significantly with tamoxifen, with durable control of her acromegaly and breast cancer. This case highlights the dual benefit of tamoxifen therapy in the treatment of hormone-receptor-positive breast cancer and acromegaly. Unlike anastrozole, tamoxifen has the benefit of lowering IGF-1 levels, which underscores its advantage in reducing adverse musculoskeletal symptoms during the treatment of hormone-receptor positive breast cancer. Additionally, tamoxifen offers an additional, oral treatment option for patients with acromegaly who might not achieve biochemical targets or cannot tolerate conventional medical therapy (such as somatostatin analogs or growth hormone receptor antagonists), though additional studies are necessary.

## Data Availability

Not applicable.

## Abbreviations

ER: Estrogen receptor
PR: Progesterone receptor
GH: Growth hormone
IGF-1: Insulin-like growth factor 1
SERMs: Selective estrogen receptor modulators
